# Method of Making Crown for a Difficult Case

**Published:** 1904-10

**Authors:** C. Frederick Rogers

**Affiliations:** Conneaut, Ohio.


### METHOD OF MAKING CROWN FOR A DIFFICULT CASE.

By C. Frederick Rogers, D. D. S., Conneaut, Ohio.

(Written for *The American Journal of Dental Science.*)

We often are called upon to crown a tooth, especially cuspids, the roots of which are exposed, labially, the gum having receded probably from 1/8 to 1/4 inch. A Richmond crown is not especially desirable, and any of the factory crowns are out of the question, for there would be more grinding to be done lingually than they would stand, even if one could be bought that would have sufficient length. The following method is especially adapted to these cases but can not be improved on for any case of crowning. After grinding the root well under the gum line, and selecting and grinding a bridge facing to accurately fit at the neck, cut a piece of 1-1000 platinum foil large enough to cover the end of the root, pressing the same well against the root with a cylinder of vulcanite rubber. This will not only give you an accurate impression of the root, but also the outline. Make a little cut with a sharp knife over the dowell hole in root, and lay aside. Fit a platinum pin loosely in root. Remove dowell from root and lightly press the pins in facing around dowell, just enough to hold them together. Place facing with dowell attached on root and after adjusting the facing in proper relation to root and while holding same in place, press the pins tightly against dowell with a pair of pliers, remove all together and set pin in a hole in fire clay furnace slab. Cut a very small wedge of pure gold, and wedge same in between dowell and pins next to the facing; place all in the furnace and solder. Bring heat quite high after gold flows to thoroughly incorporate gold in platinum. After cooling slowly, place disk of 1-1000 platinum, already prepared on end of root, melt quite a little ball of sticky wax on the facing and dowell, and force all against the platinum. Remove all together, chill wax, and press pin clear to the platinum in Mellot's Mouldine. Place in the furnace, and as the wax melts, wipe off with a piece of cotton, bring heat to a point where wax is entirely burned out. An investment other than Mouldine, would not be objectionable, if one wished, but after using plaster, Sump. asbestos, etc., I prefer Mouldine. After cooling, mix Brewster's foundation body quite thin and pack well between neck of facing and platinum, not building up too much for the first bake, or you will be cut off from the neck. After baking to biscuit, build well around the pin, giving contour required. Bake this time good and hard, as in this baking depends the strength of the crown. Be sure and bake thoroughly.

After again cooling, build on enamel body of proper color to the contour desired and bake to glaze. When cool, strip off the platinum foil, and you will have a tooth that is as perfect as human hands can make it, with as much or more strength than can be had in any crown.

About one and a half hours is required to properly prepare the root and bake the crown. Some may object to so long a time, but this crown has been such a satisfaction to me, and the fit at the neck so far away from any guess work that I have almost entirely discarded all other crowns. If one wishes, the patient can leave the chair while backing is baked, and most find this a very agreeable change.

---

### THE INTERCHANGE OF STATE CERTIFICATES.

By Dr. H. G. Patterson, Boise, Idaho, President of the State Board of Dental Examiners.

(Written for *The American Journal of Dental Science.*)

